# Empowering teachers through school digital transformation: the mediating role of digital resilience and the moderating effect of computational thinking on teachers’ digital competence

**DOI:** 10.3389/fpsyg.2026.1868343

**Published:** 2026-10-14

**Authors:** Shan Zhang, Qi Wang, Xuchen Yu, Xue Lu

**Affiliations:** 1 Faculty of Artificial Intelligence Education, Central China Normal University, Wuhan, China; 2 School of Education, Huanggang Normal University, Huanggang, China

**Keywords:** computational thinking, conservation of resources, digital resilience, school digital support, teachers’ digital competence

## Abstract

While school digital infrastructure has expanded rapidly, how institutional support translates into teachers’ digital competence remains insufficiently understood. Grounded in the Conservation of Resources theory, this study examined how digital resilience mediates the relationship between school digital support and teachers’ digital competence, and whether computational thinking moderates these associations. Data were collected from 466 Chinese educators and were analyzed using multiple regression, together with mediation and moderation analyses. The results indicate that school digital support is positively associated with teachers’ digital competence; digital resilience serves as a significant mediator in this process; moreover, computational thinking moderates the second-stage path from digital resilience to teachers’ digital competence; consequently, the indirect effect of school digital support on teachers’ digital competence via digital resilience varies across levels of computational thinking.

## 1 Introduction

Digital technologies are increasingly integrated into educational settings, with the expectation that they can support students’ problem-solving skills, connect learning to real-world contexts, and support collaboration and engagement (Falloon, 2020; Peled, 2021). For teachers, this integration makes digital competence a core professional requirement. Teachers’ digital competence refers to educators’ ability to use digital technologies effectively for instructional design, student engagement, assessment, and professional development (Karakose et al., 2021; Siddiq et al., 2016). It involves not only the operational skills needed to use digital tools, but also the pedagogical judgment required to select high-quality resources, diversify curricular designs, and adapt teaching to learner differences (Hew, 2016; Engeness, 2021; Rolf et al., 2019). Although schools worldwide have invested heavily in digital infrastructure, the availability of technological resources does not automatically translate into improved teachers’ digital competence (Gil-Flores et al., 2017).

To address this question, we draw on the Conservation of Resources (COR) theory, which holds that individuals are motivated to conserve and accumulate resources to meet environmental demands (Liu et al., 2026). COR theory distinguishes between conditional resources, such as school digital support and technological infrastructure, and personal resources, such as psychological and cognitive capacities. When individuals perceive adequate external resources, they are more likely to develop internal personal resources that support adaptive functioning (Liu, 2024; Shen et al., 2024). In this study, we treat digital resilience as one such personal resource. Digital resilience refers to teachers’ ability to maintain emotional stability, sustain adaptive functioning, and recover from setbacks when the technological environment changes (Pozo-Rico et al., 2023). Empirical studies show that resilience is positively associated with teachers’ digital competence (Shi et al., 2025). School digital support may provide teachers with the contextual resources needed to build digital resilience; teachers with higher digital resilience, in turn, may be more willing to explore the pedagogical potential of technology and adapt their instructional approaches (Collie et al., 2012).

Furthermore, COR theory suggests that the presence of complementary personal resources can influence the efficiency of resource investment and gain. Accordingly, this study examines whether teachers’ computational thinking operates as a potential moderator in this process. Computational thinking is widely defined as a problem-solving process encompassing problem decomposition, pattern recognition, abstraction, and algorithmic reasoning (Wing, 2006). In this study, we focus on four cognitive dimensions most relevant to instructional decision-making-problem decomposition, pattern recognition, abstraction and generalization, and algorithmic reasoning-while excluding sub-dimensions such as debugging and automation, as the latter are less closely related to teachers’ daily instructional practices (Grover and Pea, 2013; Liu et al., 2024). Teachers with stronger CT abilities may be better able to translate digital resilience into digital competence, as CT enables them to analyze digital teaching challenges and develop effective solutions.

Existing studies have mostly examined school digital support, digital resilience, and computational thinking as separate predictors of teachers’ digital competence; few have tested how these factors work together within one model. Grounded in COR theory, this study examines how school digital support, digital resilience, and computational thinking work together to shape teachers’ digital competence. In this model, school digital support is the predictor, teachers’ digital competence is the outcome, digital resilience is the mediator, and computational thinking is the second-stage moderator of the digital resilience to teachers’ digital competence path. Specifically, we address three research questions:

*RQ1*: How is school digital support associated with teachers’ digital competence?

*RQ2*: How does digital resilience function in the relationship between school digital support and teachers’ digital competence?

*RQ3*: Does computational thinking moderate the relationship between digital resilience and teachers’ digital competence, thereby conditioning the indirect relationship between school digital support and teachers’ digital competence through digital resilience?

## 2 Theoretical basis and framework

Several studies have investigated the pedagogical digital competencies of various groups, including university student-teachers (Jiménez-Hernández et al., 2020; Pozas and Letzel, 2023), secondary school educators (Kalinga and Ndibalema, 2023; Mtebe, 2020), and tutors in teacher training institutions (Lubuva et al., 2022). These studies have utilized different ICT competency frameworks to evaluate the digital competence of teachers, such as Technological Pedagogical Content Knowledge (TPACK) and ICT Competency Framework for Teachers (ICT CFT) (Kalinga and Ndibalema, 2023; Mtebe, 2020; Lubuva et al., 2022). A significant finding across these studies is that teachers generally view digital technologies as beneficial for pedagogical purposes. While some research has utilized the ICT Competency ICT CFT to assess the digital competencies of educators in China, there remains a need for a more thorough understanding of these competencies as guided by the ICT CFT.

### 2.1 UNESCO ICT CFT and teachers’ digital competence

This research is founded on UNESCO’s ICT Competency Framework for Teachers (ICT CFT), a globally acknowledged resource designed to assist in the training and evaluation of educators regarding their use of digital technologies in educational settings (Bitegeko et al., 2024). The ICT CFT organizes three levels and six dimensions of practice of teachers’ competence in the use of ICT in teaching and learning, culminating in the construction of 18 abilities for the application of information technology in education. Teachers’ digital competence is an important component of ICT CFT. Digital competence refers to the proficient utilization of digital knowledge, skills, and mindsets (Tondeur et al., 2017). This framework is suited to the present study because it explicitly embeds institutional support (e.g., school digital infrastructure) as a prerequisite condition for competence development at the knowledge acquisition level, thereby offering a structural anchor for examining how external provisions translate into individual capability.

Integrating COR theory with the ICT CFT offers a complementary perspective on how external provisions relate to teachers’ digital competence development (Caena and Redecker, 2019). From a COR perspective, the teachers’ digital competence described by DigCompEdu can be viewed as an adaptive outcome resulting from the joint operation of personal characteristic resources and energy resources, whereas school digital support, technological infrastructure, and professional development opportunities correspond to conditional resources. According to the resource gain principle of COR theory, when teachers perceive adequate conditional resources, they are more likely to develop internal psychological resources (e.g., digital resilience) and cognitive resources (e.g., computational thinking) that support adaptive functioning; these personal resources, in turn, are associated with higher teachers’ digital competence (Shi et al., 2025).

### 2.2 School digital support and teachers’ digital competence

School digital support brings together a range of technologies, platforms, and tools, as well as including implicit systems, climates, and cultures to support the emergence and development of teachers’ digital competence. Systematic support structures, encompassing targeted faculty development initiatives (Abdul Razzak, 2015; Hur et al., 2016) and institutional policies facilitating educational digitalization (Agyei and Voogt, 2014; Newland and Handley, 2016), serve as critical catalysts in enabling educators to integrate innovative technological pedagogies into curriculum design.

As far as teacher education and teaching are concerned, the ability of teachers to wisely choose digital tools in the digital work environment to facilitate student learning is a very critical issue (Pettersson, 2018); in addition, prior research has discussed teachers’ digital competence is closely related to the efficiency of the work performance of all departments in the digital work environment (Tabieh et al., 2021; Eyal, 2012). From the opposite perspective, related research suggests that the lack of technology in digital work environments is a major barrier to the development of digital competence among teachers. The diverse use of digital tools is associated with higher teachers’ digital competence in a digital workplace environment (Chen, 2021). Research has indicated that teachers who utilize a greater variety of digital tools tend to report more comprehensive digital competencies (Ghomi and Redecker, 2019).

### 2.3 Digital resilience and teachers’ digital competence

Digital resilience represents a burgeoning concept evolving alongside the advancement of digital education and work landscapes. Conceptually, it encapsulates the competence to preserve emotional homeostasis and deploy adaptive coping mechanisms when confronted with disruptions induced by technological innovation and the complexities of immersive digital contexts. Digital resilience, as an integrative adaptive capacity encompassing metacognitive awareness, contextual acuity, self-efficacy beliefs, and strategic regulatory skills, helps educators to navigate technological disruptions, accommodate evolving digital skill demands, and sustain constructive engagement amidst digital transformation (Mcdonnell et al., 2022; Lee and Hancock, 2023).

Grounded in COR, digital resilience functions as a core resource preservation and investment mechanism that mediates the translation of institutional digital affordances into individual professional capabilities. When schools provide robust digital support through stable platform access, systematic professional development, and responsive technical assistance, teachers are more likely to develop and sustain high levels of digital resilience; that is, the capacity to maintain efficacy beliefs, rapidly recover from setbacks, and persist in constructive pedagogical actions when confronting technological malfunctions, platform iterations, or instructional paradigm shifts (Amoako and Anane, 2025). Digital resilience may thus buffer resource depletion and support teachers in converting institutional support into instructional capability (Pozo-Rico et al., 2023).

### 2.4 Computational thinking and teachers’ digital competence

Computational thinking is one of the newly identified core competencies for the 21st century (Ainley et al., 2016; Wing, 2008). Computational thinking comprises a set of both foundational and cross-disciplinary skills that empower individuals to tackle intricate challenges, tasks, and issues arising from digitalization through the utilization of digital media. It is one of the emerging trends that make up digital competence (Schreiner and Wiesner, 2023). The essence of digital competence lies in enhancing individual digital technology operation skills and developing unique thinking styles of using digital technology to solve problems, known as computational thinking. Some studies have indicated a positive relationship between digital competence and computational thinking (Esteve-Mon et al., 2020).

Within ICT CFT framework, computational thinking is classified as a technology-specific competency distinct from general digital competence, representing a personal cognitive resource that affects how individuals acquire, utilize, and benefit from contextual resources such as school-based digital support. Specifically, COR theory distinguishes between resource gain spirals and resource loss cycles, and suggests that the possession of key personal resources influences the extent to which individuals can effectively draw upon external resources to prevent depletion and promote gain. This theoretical principle aligns closely with conceptualizing computational thinking as a moderating variable. As a higher-order metacognitive capability, computational thinking functions to moderate the second-stage resource-translation process: when teachers possess higher levels of computational thinking, they can more effectively translate digital resilience into teachers’ digital competence.

Based on previous research, this study proposes five hypotheses:

*H1*: School digital support is positively associated with teachers’ digital competence.

*H2*: Digital resilience is positively associated with teachers’ digital competence.

*H3*: School digital support is positively associated with digital resilience.

*H4*: Digital resilience mediates the association between school digital support and the formation of teachers’ digital competence.

*H5*: Computational thinking moderates the relationship between digital resilience and teachers’ digital competence (the second-stage path), thereby conditioning the indirect relationship between school digital support and teachers’ digital competence through digital resilience.

## 3 Materials and methods

### 3.1 Participants

This study adopts a quantitative case study design, involving the collection and statistical analysis of survey data from participating teachers. A stratified random sampling strategy was employed to ensure representativeness across different educational strata. Participating teachers were selected from primary and secondary schools in three provinces of central China, covering four tiers of administrative regions: provincial capitals, prefecture-level cities, county-level cities, and rural townships. Strata were defined by school location and educational level (primary vs. secondary). Within each stratum, schools were randomly selected, and teachers were then randomly selected from within those schools.

A total of 500 questionnaires were distributed, of which 482 were returned, yielding a response rate of 96.4%. After applying stringent quality control filters-excluding submissions with excessively short completion times (less than 0.2 s per item), significant item non-response, logically inconsistent demographic details, or invariant response patterns-466 valid questionnaires remained. Non-response bias was assessed by comparing early and late respondents on key demographic variables, and no significant differences were found, suggesting that non-response bias is unlikely to pose a serious threat to the validity of the findings.

This study underwent review and received approval from the Institutional Review Board (IRB) of the university where the research was conducted. The IRB determined that the study posed minimal risk to participants. Prior to questionnaire administration, all participants received a detailed explanation of the research purpose, procedures, and their rights, including privacy protection and the voluntary nature of participation. The research objectives were clearly stated, and the commitment to ensuring the anonymity and confidentiality of participants’ information and research findings was emphasized. All collected data were used exclusively for research purposes, stored securely, and accessible only to the research team.

### 3.2 Instruments

#### 3.2.1 School digital support

The School Digital Support Scale was adapted from the DigCompEdu Check-In questionnaire (Ghomi and Redecker, 2019) to assess teachers’ perceived access to institutional resources facilitating technology integration. Conceptually, school digital support refers to the systematic provision of technological infrastructure, professional development, and organizational arrangements that constitute a conducive digital work environment for teachers (Karakose et al., 2021).

The adaptation followed a multi-stage procedure. First, the Chinese version was established through forward translation, blind back-translation, and reconciliation by an expert panel. Second, content validity was evaluated, yielding I-CVI values of 0.86–1.00 and an S-CVI of 0.94. Third, a pilot study with 62 in-service teachers was conducted, with two items refined accordingly. Fourth, EFA extracted two factors explaining 77.79% of the variance, with all items loading saliently on their hypothesized factors. Fifth, CFA confirmed the two-factor structure, with standardized factor loadings of 0.795–0.921, AVE = 0.764, and CR = 0.958.

The final instrument comprises eight items measuring two dimensions. Dimension 1, diversity of digital tools provision, modified the original item “I can employ interactive whiteboards, projectors, or presentation software in classroom instruction” to “My school provides diverse digital tools for classroom instruction” to capture organizational resource availability rather than individual self-efficacy. Dimension 2, availability of school-based digital training, retained the original item core with minor linguistic adaptation. All items were rated on a 5-point Likert scale (1 = strongly disagree, 5 = strongly agree), with higher scores indicating greater perceived school digital support.

#### 3.2.2 Teachers’ digital competence

The Teachers’ digital competence Scale was adopted from the Chinese localized version of the DigCompEdu framework (Li et al., 2024) to assess primary and secondary school teachers’ self-perceived digital competence. Conceptually, this construct denotes the self-assessed capacity to proficiently utilize digital knowledge, pedagogical mindsets, and strategic decision-making skills across six domains: professional engagement, digital resources, teaching and learning, assessment, empowering learners, and facilitating learners’ digital competence. These items are divided into six dimensions: Professional Engagement (encompassing items numbered 1_1 to 1_4); Digital Resources (covering items 2_1 to 2_3); Teaching and Learning (spanning items 3_1 to 3_4); Assessment (comprising items 4_1 to 4_3); Empowering Learners (including items 5_1 to 5_3) and Facilitating Learners’ Digital Competence (ranging from items 6_1 to 6_5).

Notably, the initial CFA for the Facilitating Learners’ Digital Competence dimension yielded an AVE of 0.486, marginally below the conventional threshold. Expert review identified one item with ambiguous construct boundaries-conflating digital content creation with online ethics-that exhibited a low factor loading and non-negligible cross-loadings. Following its removal, the model was re-estimated with four remaining items, producing standardized factor loadings of 0.827–0.892, an AVE of 0.737, and a CR of 0.918. These values satisfy the stringent convergent validity criterion and confirm the dimension’s psychometric adequacy.

The scale employs a self-assessment descriptive rubric rather than a traditional Likert format, with each item scored from 1 to 5 anchored to specific behavioral criteria denoting progressive mastery. An example item is provided below:

*I can create digital resources (such as inserting video resources in PPT, recording micro-lessons, and screen recording), and modify them to meet my instructional needs.*

*The options are as follows:*

*I cannot create digital resources. (1 points).*

*I can indeed use a computer to make handouts or reading lists, but I still prefer to print them out. (2 points).*

*I can only make PowerPoint presentations on the computer. (3 points).*

*I can create and modify different types of digital resources. (4 points).*

*I can conduct lessons using various resources, such as multimedia presentations and online materials. (5 points).*

#### 3.2.3 Digital resilience

The Digital Resilience Scale was adapted from Makrakis (2024), an instrument developed and validated specifically for teachers working with ICT-based learning technologies, to assess educators’ technology-specific stress-coping strategies in the context of educational technology integration. The decision to focus on this facet of digital resilience constitutes a deliberate delimitation of the study rather than an incidental restriction, and it is grounded in three considerations. First, it follows from the construct definition adopted in this study, in which digital resilience refers to teachers’ ability to maintain emotional stability, sustain adaptive functioning, and recover from setbacks when the technological environment changes (Pozo-Rico et al., 2023); the scale’s focus on regulating negative affect, tolerating technological setbacks, and restoring instructional efficacy maps onto this definition. Second, digital resilience is increasingly conceptualized as a domain-specific capacity rather than a general trait (Lee and Hancock, 2023), and the present research questions concern teachers’ functioning specifically in digital teaching contexts; incorporating broader, non-technological facets of resilience would therefore introduce construct contamination rather than improve coverage. Third, adapting a validated, context-matched instrument preserves measurement fidelity more effectively than assembling items across general resilience scales, and the psychometric evidence reported below supports the adequacy of this operationalization. Accordingly, the scale specifically targets teachers’ capacity to regulate negative affect, tolerate technological setbacks, and restore instructional efficacy when confronted with technical difficulties, platform changes, or instructional uncertainty.

Two bilingual researchers independently translated items into Chinese, followed by a blind back-translation. Discrepancies were resolved by an expert panel (three educational psychology professors and one teacher education specialist). The panel ensured each item targeted a single technology-induced stress context, yielding I-CVI of 0.88–1.00 and S-CVI of 0.96. A pilot study with 52 teachers confirmed item discrimination. EFA supported a unidimensional structure; CFA factor loadings of 0.765–0.939, AVE = 0.792, and CR = 0.938.

This four-item instrument assesses educators’ digital resilience in the face of digital disruptions (sample item: “When digital teaching tools malfunction unexpectedly, I remain composed and persist in seeking alternative solutions”). All items are rated on a 5-point Likert scale anchored at 1 (strongly disagree) and 5 (strongly agree), with higher scores indicating greater digital resilience in coping with technology-induced stress.

#### 3.2.4 Computational thinking

The Computational Thinking Scale was adapted from Yin et al. (2020) to assess these pedagogically oriented manifestations of computational thinking in digitally mediated instructional contexts. Rather than measuring the full spectrum of the construct, the scale was deliberately narrowed to four core dimensions most proximal to teaching: problem decomposition, pattern recognition, abstraction and generalization, and algorithmic reasoning. Sub-dimensions such as debugging and automation were excluded during adaptation because they are less directly implicated in routine pedagogical decision-making. Consequently, the items reflect pedagogical problem-solving and self-perceived cognitive competence in instructional contexts rather than computational thinking in its broader theoretical sense. We explicitly acknowledge that this operationalization prioritizes pedagogical applicability over comprehensive construct coverage, and that future research may benefit from incorporating a fuller range of computational thinking dimensions to validate the generalizability of the present findings.

The adaptation followed a multi-stage procedure. First, two bilingual researchers independently translated the items into Chinese, followed by blind back-translation and reconciliation by an expert panel. Second, the panel evaluated content validity, ensuring items captured specific cognitive operations rather than general problem-solving self-efficacy (I-CVI = 0.80–1.00; S-CVI = 0.95). Third, a pilot study with 52 in-service teachers confirmed item comprehensibility and adequate discrimination. Fourth, confirmatory factor analysis validated the unidimensional structure, with standardized factor loadings ranging from 0.896 to 0.943, AVE of 0.858, and CR of 0.980.

The final eight-item instrument measures the four theoretically specified dimensions. Example items include: “I am capable of decomposing intricate problems into simpler, more understandable components” (problem decomposition) and “I can identify recurring patterns in students’ digital learning behaviors to inform my instructional adjustments” (pattern recognition). All items were rated on a 5-point Likert scale ranging from 1 (not able to perform the task) to 5 (fully capable of performing the task), with higher scores indicating stronger computational thinking in pedagogical problem-solving contexts.

To ensure that the four core constructs are empirically distinct, discriminant validity was assessed using two complementary procedures. First, the Fornell–Larcker criterion was applied by comparing the square root of AVE for each construct with its bivariate correlations with other constructs. As shown in Table 1, the square root of AVE for each construct exceeds its correlations with all other constructs (off-diagonal values), satisfying the stringent requirement for discriminant validity.

**Table 1:** Fornell–Larcker criterion.

	1	2	3	4
1. School digital support	**0.847**			
2. Digital resilience	0.649	**0.890**		
3. Computational thinking	0.460	0.412	**0.926**	
4. Teachers’ digital competence	0.470	0.427	0.472	**0.810**

The bold values on the diagonal are the square roots of AVE; the values off the diagonal are Pearson correlations.

Second, the heterotrait–monotrait ratio of correlations was computed based on the factor loadings and construct correlations. All HTMT values range from 0.41 to 0.64, well below the conservative threshold of 0.85, confirming that the constructs are not interchangeable.

Since this study relies on self-reported data from respondents and the measurement environment is not under the subjective control of the researcher, there is a potential risk of common method bias (CMB). To minimize the impact of common method variance (CMV), multiple control measures were implemented in both the data collection and data analysis stages. During data collection, we employed anonymous questionnaires, included lie-detection items, and randomized the order of questions to reduce subjective bias from respondents. In the data analysis phase, Harman’s single-factor test was conducted to assess the presence of CMB. The results showed that the largest single factor accounted for only 38.522% of the total variance, which is below the commonly used threshold of 40% that indicates significant common method variance (Podsakoff et al., 2003). These findings suggest that CMB is unlikely to pose a serious threat to the validity of the study’s conclusions.

#### 3.2.5 Control variables

This study incorporates several relevant covariates, including teachers’ gender, years of teaching experience and educational level. We treat teachers’ gender as a control variable, as it may impact their technological competence, with studies indicating that female teachers are generally less inclined to utilize technology in their teaching (Gil-Flores et al., 2017). Considering the inverse relationship found between years of teaching experience and the successful integration of technology in education (Hsu et al., 2017), we also included this variable as a control. Additionally, teachers’ educational level is regarded as a control variable as well, with research suggesting that higher educational attainment among teachers correlates with improved competence in integrating information technology into their teaching practices (Badia et al., 2017).

### 3.3 Analysis procedure

We analyzed the data using IBM SPSS version 27.0. First, descriptive statistics were computed to examine the distribution of the data and the characteristics of the variables, followed by correlation analyses among the study variables. Mediation and moderated mediation effects were tested using the PROCESS macro for SPSS developed by Hayes (2013). Following Preacher and Hayes (2008), we applied a bias-corrected percentile bootstrap with 5,000 resamples to estimate the indirect effect and its confidence intervals.

## 4 Results

### 4.1 Descriptive analyses and correlation analyses

The demographic characteristics of these participants are summarized in Table 2. In terms of gender distribution, 27.9% of the respondents identified as male (130), while 72.1% identified as female (336). Regarding the geographic distribution of the teachers’ schools, 136 teachers (29.2%) were from urban areas, while 330 teachers (70.8%) were from rural settings. The composition of respondents according to their teaching experience was as follows: 97 teachers (20.8%) had 5 years or less of experience, 59 teachers (12.7%) had 6–10 years, 45 teachers (9.7%) had 11–15 years, 37 teachers (7.9%) had 16–20 years, 79 teachers (17.0%) had 21–25 years, and 149 teachers (32.0%) had more than 25 years of experience. In terms of educational qualifications, 1.7% held a master’s degree (*n* = 8), 76.2% possessed a bachelor’s degree (*n* = 355), and 22.1% had a college degree or lower (*n* = 103). Relative to nationwide figures indicating that male teachers account for roughly 29% of primary school faculty in China, this investigation revealed a lower representation at 27.9%. Concurrently, educational credential compliance was observed in 99% of respondents, aligning closely with national benchmarks.

**Table 2:** Demographic characteristics of the respondent (*N* = 466).

Variate	Category	*N*	Percent (%)
Gender	Male	130	27.9
	Female	336	72.1
Location	Rural	330	70.8
	Urban	136	29.2
Teaching experience (year)	5 years or less	97	20.8
	6 ~ 10	59	12.7
	11 ~ 15	45	9.7
	16 ~ 20	37	7.9
	21 ~ 25	79	17.0
	Over 25 years	149	32.0
Level of education	Not attend college	7	1.5
	Undergone 3 years at college	96	20.6
	Bachelor’s degree	355	76.2
	Master’s degree	8	1.7

Table 3 presents the descriptive statistics and correlation matrix for the study’s key constructs. All variables were assessed using a 5-point Likert-type scale, with the theoretical midpoint of 3.0 serving as the interpretive benchmark. As shown in Table 3, computational thinking exhibited the highest mean score (*M* = 3.770, *SD* = 0.797), reflecting participants’ generally favorable self-assessment of their capabilities in problem decomposition, pattern recognition, abstraction and generalization, and algorithmic reasoning within instructional contexts. School digital support registered a mean above the scale midpoint (*M* = 3.390, *SD* = 0.753), suggesting moderate-to-favorable perceptions of institutional resource provision. Digital resilience demonstrated a comparable mean (*M* = 3.335, *SD* = 0.875), indicating moderate self-reported capacity to cope with technological demands and uncertainties. By contrast, teachers’ digital competence exhibited the lowest mean (*M* = 2.867, *SD* = 0.783), registering marginally below the scale midpoint; this suggests a relatively modest level of self-assessed proficiency in integrating digital technologies into instructional practice. Consistent with expectations, there is a positive and significant correlation among all the variables under study. The skewness and kurtosis values indicate that the distribution of all research variables is close to normal, with skewness values within the range of |2.0| and kurtosis values within |7.0|, which aligns with the criteria set by Hancock et al. (2018).

**Table 3:** Descriptive statistics and correlation matrix of research variables.

	1	2	3	4
1. School digital support	1			
2. Digital resilience	0.649***	1		
3. Teachers’ digital competence	0.460***	0.412***	1	
4. Computational thinking	0.470***	0.427***	0.472***	1
*M*	3.390	3.335	2.867	3.770
*SD*	0.753	0.875	0.783	0.797
*Skewness*	0.562	0.096	0.639	−0.966
*Kurtosis*	−0.093	0.195	0.054	1.928

***p < 0.001, **p < 0.01, *p < 0.05.

### 4.2 The mediating function of teachers’ digital resilience

To ensure the robustness of our analytical framework, we conducted comprehensive diagnostic procedures before the moderated mediation analysis. Normality of disturbances across the six digital competence dimensions was confirmed via P–P plot inspections, which indicated that error terms followed a normal distribution. Tolerance values ranged between 0.533 and 0.754 (exceeding the 0.1 threshold), while Variance Inflation Factors remained below 1.857 (substantially under the conventional cutoff of 10). Additionally, the condition index of 14.801 fell well below the critical value of 15. Collectively, these findings affirm that collinearity does not threaten model validity, providing a statistically sound foundation for interpreting the subsequent moderated mediation results.

To account for potential confounding effects, we included gender, education level and years of teaching experience as covariates in the regression models. The results of this analysis are presented in Table 4. The results supporting Hypothesis 1 reveal that the school digital support significantly predicts teachers’ digital competence, with a path coefficient of (*B* = 0.459, *t* = 10.637, *p* < 0.001). School digital support was also positively associated with digital resilience (*B* = 0.739, SE = 0.041, *t* = 17.909, *p* < 0.001), supporting Hypothesis 3. When digital resilience was added, the direct association between school digital support and teachers’ digital competence remained significant (*B* = 0.343, SE = 0.056, *t* = 6.174, *p* < 0.001).

**Table 4:** Analysis of school digital support, digital resilience, and teachers’ digital competence.

	Step1: TDC			Step2: DR			Step3: TDC		
	*B*	*SE*	*t*	*B*	*SE*	*t*	*B*	*SE*	*t*
Constant	1.584	0.308	5.142^***^	0.839	0.295	2.846^**^	1.453	0.308	4.724^***^
Gender	−0.103	0.075	−1.367	−0.088	0.072	−1.228	−0.089	0.074	−1.194
Teaching experience	−0.009	0.003	−3.021^**^	−0.007	0.003	−2.409^*^	−0.008	0.003	−2.672^**^
Level of education	0.026	0.069	0.382	0.098	0.066	1.484	0.011	0.068	0.162
SDS	0.459	0.043	10.637^***^	0.739	0.041	17.909^***^	0.343	0.056	6.174^***^
DR							0.156	0.048	3.245^**^
*R*	0.480			0.660			0.497		
*R* ^2^	0.230			0.435			0.247		
*F*	34.432^***^			88.618^***^			30.221^***^		

SDS, school digital support; TDC, teachers’ digital competence; DR, digital resilience. ***p < 0.001, **p < 0.01, *p < 0.05.

Moreover, digital resilience was positively associated with teachers’ digital competence while controlling for school digital support (*B* = 0.156, SE = 0.048, *t* = 3.245, *p* = 0.001), lending further support to Hypothesis 2. The indirect effect of school digital support through digital resilience was significant (*Effect = 0.116, BootSE = 0.044, 95% BootCI [0.038, 0.210]*). The proportion mediated was 25.2%, supporting Hypothesis 4. Overall, these findings are consistent with an indirect association between school digital support and teachers’ digital competence via digital resilience.

### 4.3 The moderating function of computational thinking

After controlling for essential demographic variables, our analysis revealed a statistically significant interaction between digital resilience and computational thinking affecting teachers’ digital competence, with a path coefficient of (*B* = 0.205, *t* = 6.576, *p* < 0.001) (refer to Table 5). This pattern indicates that the second-stage path from digital resilience to teachers’ digital competence varies depending on the level of computational thinking, which is consistent with Hypothesis 5.

**Table 5:** Analysis of school digital support, digital resilience, computational thinking, and teachers’ digital competence.

	Step1: DR			Step2: TDC		
	*B*	*SE*	*t*	*B*	*SE*	*t*
Constant	0.839	0.295	2.846^**^	2.130	0.304	7.000^***^
Gender	−0.088	0.072	−1.228	−0.067	0.068	−0.985
Teaching experience	−0.007	0.003	−2.409^*^	−0.005	0.003	−1.906
Level of education	0.098	0.066	1.484	0.055	0.063	0.876
SDS	0.739	0.041	17.909^***^	0.217	0.053	4.112^***^
DR				0.083	0.045	1.862
DR × CT				0.205	0.031	6.576^***^
CT				0.366	0.043	8.544^***^
*R*	0.659			0.614		
*R* ^2^	0.435			0.377		
*F*	88.618***			39.610^***^		

SDS, school digital support; TDC, teachers’ digital competence; DR, digital resilience; CT, computational thinking. ***p < 0.001, **p < 0.01, *p < 0.05.

We utilized the SPSS Process macro to visualize the interaction effects by calculating slopes one standard deviation above and below the mean of the moderating variable, computational thinking. As depicted in Figure 1, the positive slope of digital resilience on teachers’ digital competence was steeper at higher levels of computational thinking and shallower (and non-significant) at lower levels.

**Figure 1:**
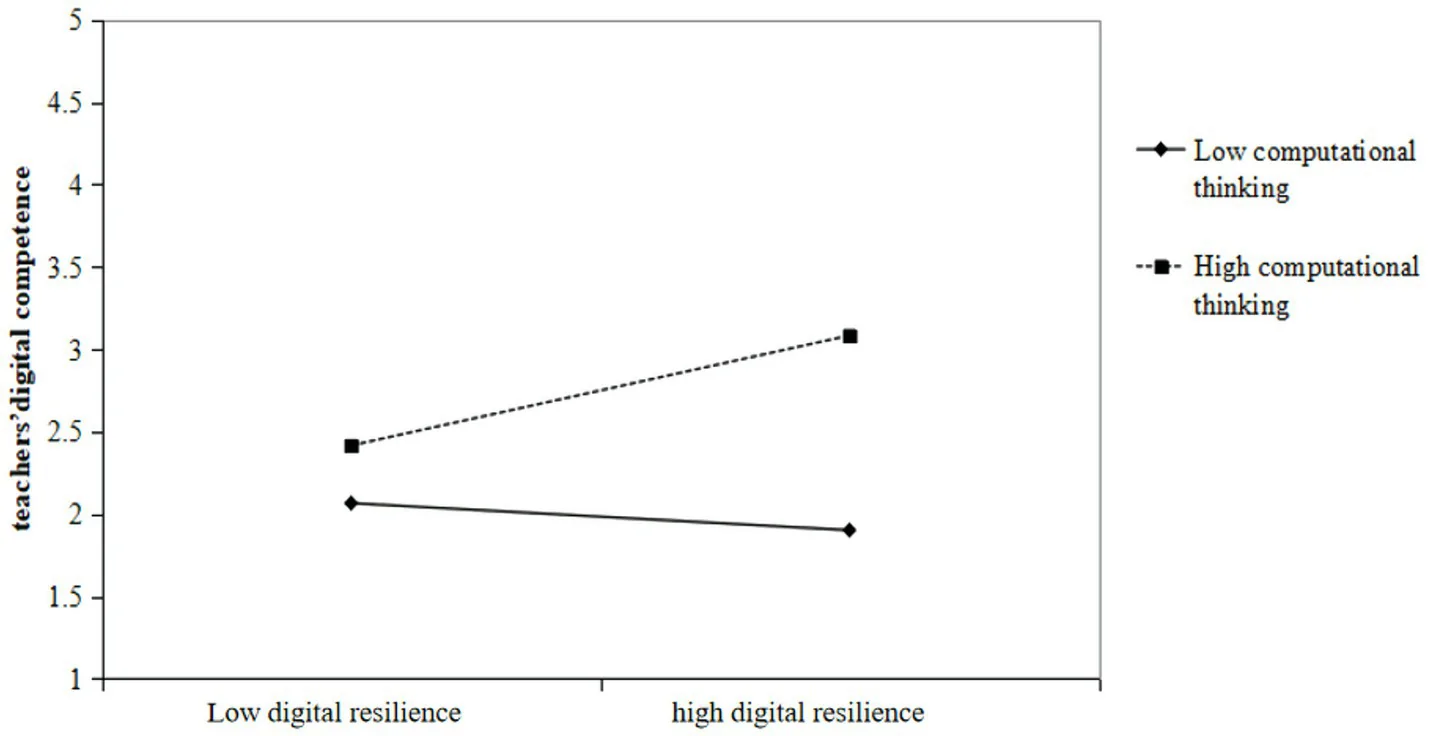
Conditional effect of digital resilience on teachers’ digital competence at low and high levels of computational thinking.

Furthermore, we tested whether the conditional indirect effect of school digital support on teachers’ digital competence through digital resilience varied across levels of computational thinking. As shown in Table 6, the conditional indirect effect was not significant at one standard deviation below the mean of computational thinking (*Effect = −0.059, BootSE = 0.048, 95%BootCI [−0.150, 0.037]*) and was not significant at the mean (*Effect = 0.062, BootSE = 0.039, 95%BootCI [−0.010, 0.145]*), because both bootstrap confidence intervals included zero. The conditional indirect effect was significant only at one standard deviation above the mean of computational thinking (*Effect = 0.182, BootSE = 0.049, 95%BootCI [0.093, 0.283]*). The index of moderated mediation was significant (*Index = 0.152, BootSE = 0.035, 95% BootCI [0.083, 0.218]*).

**Table 6:** Conditional indirect effects of school digital support via digital resilience at levels of computational thinking.

	SDS	Effect	Boot SE	Boot low CI	Boot high CI
Level of CT	M−1SD	−0.059	0.048	−0.150	0.037
	M	0.062	0.039	−0.010	0.145
	M + 1SD	0.182	0.049	0.093	0.283

CT, computational thinking.

## 5 Discussion and implications

Although extensive research has examined the relationship between school digital support and teachers’ digital competence (Abdul Razzak, 2015; Gil-Flores et al., 2017), the underlying mechanisms of this association remain insufficiently understood. This research investigates a moderated mediation model to clarify the relationship between school digital support and teachers’ digital competence. The results indicate that school digital support was positively associated with teachers’ digital competence in the total-effect model (*B* = 0.459, *t* = 10.637, *p* < 0.001). Additionally, school digital support was positively associated with digital resilience (*B* = 0.739, *t* = 17.909, *p* < 0.001), which in turn was positively associated with teachers’ digital competence when school digital support was controlled (*B* = 0.156, *t* = 3.245, *p* = 0.001).

Consistent with RQ2, the findings reveal a significant indirect association between school digital support and teachers’ digital competence via digital resilience (*indirect effect = 0.116, SE = 0.044, 95%CI [0.038, 0.210]*). School digital support may be linked to teachers’ digital competence in part through fostering their capacity to recover from technological setbacks and sustain adaptive engagement.

Regarding RQ3, the findings suggest that computational thinking moderates the second-stage path from digital resilience to teachers’ digital competence (*B* = 0.205, *t* = 6.576, *p* < 0.001). Thus, teachers’ computational thinking operated as a second-stage moderator, and the indirect association between school digital support and teachers’ digital competence through digital resilience was conditional on computational thinking. The conditional indirect effect was stronger at higher levels of computational thinking and reached bootstrap significance only at one standard deviation above the mean; it was not significant at the mean or at one standard deviation below the mean.

### 5.1 Theoretical implications

The findings address the three research questions as follows, with interpretations bounded by the scope and delimitations of this study. Regarding RQ1, according to the study outcomes, school digital support and teachers’ digital competence show a significantly positive correlation in our sample. Grounded in the COR theory, conditional resources are linked to the accumulation of personal resources, which in turn correspond to favorable professional outcomes. Specifically, prior work suggests that technical infrastructure and supportive institutional environments may help teachers respond to the opportunities and challenges presented by digital pedagogical models (Yurinova et al., 2022).

Regarding RQ2, according to the study outcomes, school digital support and digital resilience show a significantly positive correlation, as do digital resilience and digital competence. In our data, the bootstrap confidence interval for the indirect effect did not include zero, which is consistent with a mediation pathway. However, as noted in the limitations, the concurrent self-report measurement means that this mediation pattern could partially reflect shared perceptual variance rather than a purely causal process.

Regarding RQ3, the results suggest that computational thinking is associated with differences in the strength of the effect of digital resilience on teachers’ digital competence and, consequently, with variation in the conditional indirect relationship between school digital support and teachers’ digital competence through digital resilience. Prior research indicates that educators with robust computational thinking capabilities may be better equipped to respond adaptively when engaging with digital tools and technologies (Yadav et al., 2014), which may enable them to effectively navigate diverse challenges arising from technology integration and to actively explore and adopt innovative solutions (Yadav et al., 2017). However, because all variables were measured simultaneously through self-report, we cannot rule out the alternative explanation that teachers with higher self-assessed digital competence retrospectively rated both their resilience and computational thinking more favorably. Thus, while the findings are consistent with the theoretical view that computational thinking might serve as a cognitive foundation enabling teachers to leverage digital tools for instructional problem-solving (Kong et al., 2020), these interpretations remain tentative and require confirmation through longitudinal designs that can establish causal precedence and objective behavioral outcomes.

### 5.2 Practical implications

Because all constructs were measured by self-report at a single time point, the implications below concern perceived school digital support, digital resilience, computational thinking, and teachers’ digital competence; they should not be interpreted as direct evidence about objective digital performance or actual classroom behavior.

Our research findings highlight the crucial role of a digital work environment in relation to teachers’ digital competence. To support the development of teachers’ digital competence, schools may wish to prioritize the establishment of a robust digital work environment. For example, studies by Ertmer and Ottenbreit-Leftwich (2010) and Hew and Brush (2007) have shown that modernizing digital campuses can streamline essential functions such as lesson planning, teaching, homework assignments, grading, and home-school communication. School administrators may consider focusing on developing and upgrading digital teaching software and hardware to foster a connected and personalized support system for educators. Additionally, schools may fully integrate digital teaching resources from diverse sources, creating a digital resource library for teachers to support access to these materials.

Secondly, the findings of this study carry implications for teachers’ digital resilience, a factor associated with their digital competence. Schools may assist teachers in establishing clear and measurable goals for developing digital resilience, while guiding them in designing feasible implementation plans. As teachers gradually accumulate successful experiences in navigating digital instructional challenges, they may also report stronger digital resilience. Providing personalized guidance and emotional support may be associated with experience sharing and collaboration among teachers, and may be associated with less anxiety and frustration in digital instruction.

Finally, it may be beneficial to encourage teachers to actively engage in digital teaching initiatives. Through this process, teachers may have opportunities to refine their digital teaching competencies, further develop their potential, and build a stronger sense of resilience. The findings further suggest that the positive association between digital resilience and teachers’ digital competence may be more pronounced when computational thinking is higher; however, the results do not show that computational thinking strengthens the direct effect of school digital support on teachers’ digital competence. Therefore, any implication for computational-thinking training should be framed tentatively as supporting teachers’ capacity to use digital resilience within the pathway linking digital resilience to teachers’ digital competence, pending confirmation from longitudinal or experimental research (Grover and Pea, 2013).

### 5.3 Limitation and future research

Like all studies, this research has several limitations. First, the data analyzed in this study originates from the central Chinese provinces; the applicability of the conclusions to urban demographics of both the western and eastern regions of China, as well as to teachers of different areas, is subject to uncertainty. Therefore, we recommend that future researchers incorporate more diverse data sources to arrive at robust conclusions. Second, this study employed a cross-sectional design, limiting the ability to draw causal inferences. A survey like ours cannot clarify the causal relationships among school digital support, digital resilience, computational thinking, and digital competence. Future investigations could benefit from a longitudinal approach to explore causal patterns and reciprocal relationships over time.

Third, the limitations associated with the adaptation and validation of the measurement instruments require explicit discussion, as they carry direct implications for the robustness of the measurement model and the generalizability of the findings. Although pilot testing with a small in-service teacher sample was conducted for preliminary item analysis, the limited sample size likely constrained statistical power to detect problematic items. This raises the possibility that certain items with suboptimal psychometric properties, such as weak factor loadings, cross-loadings, or differential item functioning may have been retained in the main analysis, thereby affecting the precision and stability of the measurement model. More critically, the study lacks a fully independent validation sample. Without cross-validation in a separate sample, it is not possible to confirm whether the factor structure, item thresholds, and reliability estimates identified in the primary analysis are replicable or sample-dependent. This limitation reduces confidence in the measurement model’s cross-contextual generalizability and should be carefully weighed when interpreting the structural relationships reported in this study.

Last, because all core constructs were assessed through self-report at a single time point, the findings reflect teachers’ self-perceived digital competence, digital resilience, and computational thinking rather than objective performance or observed classroom behavior. Future studies could combine self-report measures with behavioral traces, performance assessments, or observer ratings.

## Data Availability

The raw data supporting the conclusions of this article will be made available by the authors, without undue reservation.
